# Expression-based GWAS identifies variants, gene interactions and key regulators affecting intramuscular fatty acid content and composition in porcine meat

**DOI:** 10.1038/srep31803

**Published:** 2016-08-18

**Authors:** Anna Puig-Oliveras, Manuel Revilla, Anna Castelló, Ana I. Fernández, Josep M. Folch, Maria Ballester

**Affiliations:** 1 Departament de Ciència Animal i dels Aliments, Universitat Autònoma de Barcelona (UAB), 08193 Bellaterra, Spain; 2 Plant and Animal Genomics, Centre de Recerca en Agrigenòmica (CRAG), 08193 Bellaterra, Spain; 3 Departamento de Genética Animal, Instituto Nacional de Investigación y Tecnología Agraria y Alimentaria (INIA), 28040 Madrid, Spain; 4 Departament de Genètica i Millora Animal, Institut de Recerca i Tecnologia Agroalimentàries (IRTA), Torre Marimon, 08140 Caldes de Montbui, Spain

## Abstract

The aim of this work is to better understand the genetic mechanisms determining two complex traits affecting porcine meat quality: intramuscular fat (IMF) content and its fatty acid (FA) composition. With this purpose, expression Genome-Wide Association Study (eGWAS) of 45 lipid-related genes associated with meat quality traits in swine muscle (*Longissimus dorsi*) of 114 Iberian × Landrace backcross animals was performed. The eGWAS identified 241 SNPs associated with 11 genes: *ACSM5, CROT, FABP3, FOS, HIF1AN, IGF2, MGLL, NCOA1, PIK3R1, PLA2G12A* and *PPARA.* Three expression Quantitative Trait Loci (eQTLs) for *IGF2, ACSM5* and *MGLL* were identified, showing *cis*-acting effects, whereas 16 eQTLs had *trans* regulatory effects. A polymorphism in the *ACSM5* promoter region associated with its expression was identified. In addition, strong candidate genes regulating *ACSM5, FOS, PPARA, PIK3R1, PLA2G12A* and *HIF1AN* gene expression were also seen. Notably, the analysis highlighted the NR3C1 transcription factor as a strong candidate gene involved in the regulation of the 45 genes analysed. Finally, the *IGF2, MGLL, MC2R, ARHGAP6*, and *NR3C1* genes were identified as potential regulators co-localizing within QTLs for fatness and growth traits in the IBMAP population. The results obtained increase our knowledge in the functional regulatory mechanisms involved in these complex traits.

Pork-meat cuts and their derived products are paid according to the lean percentage in pork carcasses and meat quality, since they determine better acceptance for consumers^1^. A high amount of backfat content is a less desirable trait; meanwhile, meat with high intramuscular fat (IMF) is considered to have better taste, conferring juiciness to the meat. Additionally, fatty acid (FA) composition of IMF affects meat nutritional and sensory quality parameters. Monounsaturated FAs (MUFA) confer more oxidative stability than do polyunsaturated FAs (PUFA), improving meat taste and colour^2^. Furthermore, PUFA decreases the risk of suffering cardiovascular diseases, being healthier than saturated FAs (SFA)^3^. Therefore, there is a consumer requirement for porcine meat with high IMF with a balanced FA composition.

Some pig breeds, such as Landrace, have undergone an intense selection process towards efficient meat production with rapid growth and a leaner carcass; however, its resulting meat is less appreciated by consumers due to having low IMF composed of a high proportion of PUFA content^4^. In contrast, rustic pigs, such as the Iberian, are fatter, with more IMF with higher SFA and MUFA content and lower PUFA concentrations, which yields high quality and tasty meat, especially for dry-cured products. For instance, when comparing Iberian pigs with commercial breeds, Iberian pigs show higher palmitoleic acid (C16:1(n-7); 4.08 vs. 3.31; p < 0.001), oleic acid (C18:1(n-9); 44.96 vs. 40.56; p < 0.001), and general MUFAs (49.08 vs. 40.25; p < 0.001), and approximately half of the linoleic acid (C18:2(n-6); 7.52 vs. 16.11; p < 0.001) proportion, which is the most abundant PUFA^4^. Moreover, Iberian pigs have more than twice the amount of IMF, when compared to Landrace pigs (1.6% vs. 3.7%)^5^. The differences in the genetic background of these breeds determine the IMF and its FA composition, affecting meat quality^5^.

The experimental IBMAP population^6^, a cross between Iberian x Landrace pigs, which clearly differs in growth, carcass and meat quality, such as the IMF content and FA composition, was generated to obtain a population with large phenotypic differences, in which alleles from both parental breeds are segregating and can be tested. Strategies such as QTL and GWAS have been useful to highlight many genes, for instance determining intramuscular fatty acid (IMFA) composition in the IBMAP population^6^,^7^,^8^,^9^,^10^,^11^,^12^,^13^. Despite this, the underlying physio-genetic complex mechanisms of IMF deposition and its FA composition have not been clarified. The difficulty in the detection of QTLs for complex traits may be influenced by the pleiotropic nature of these traits and the use of multiple-tests correction methods^14^. Integrating other types of data such as intermediate molecular phenotypes to identify associations can overcome these limitations.

The detection of expression Quantitative Trait Loci (eQTLs) has recently been proposed as a good strategy to deepen the study of the genetic architecture of complex traits^15^. This technique allows for the identification of genetic variants associated with gene transcription levels, which may determine the phenotypic differences of complex traits. With the objective to better understand the mechanisms affecting IMF content and FA composition, we performed expression Genome-Wide Association Study (eGWAS) of 45 strong candidate genes for these traits, identified in previous studies by our group^12^,^16^,^17^,^18^, in 114 BC1_LD animals.

## Results and Discussion

### Gene-expression profiling

In the present study, 114 BC1_LD animals generated from the experimental IBMAP population and showing a wide range of IMF content and FA composition values were used for RNA extraction and RT-qPCR to perform eGWAS (Supplementary Table S1).

**Figure 1:**
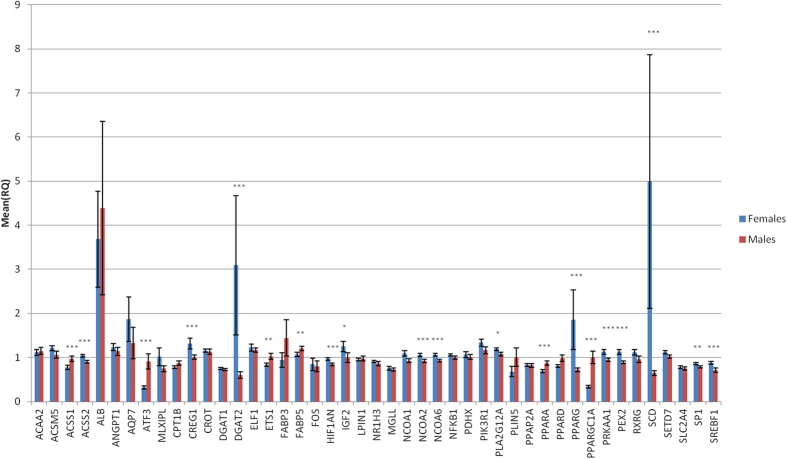
Comparison between males and females of muscle gene-expression levels of 45 lipid-related genes. Data represent means ± standard error of the means (SEM). Significant differences between sexes are indicated as *P ≤ 0.05, **P ≤ 0.01, ***P ≤ 0.001.

**Figure 2:**
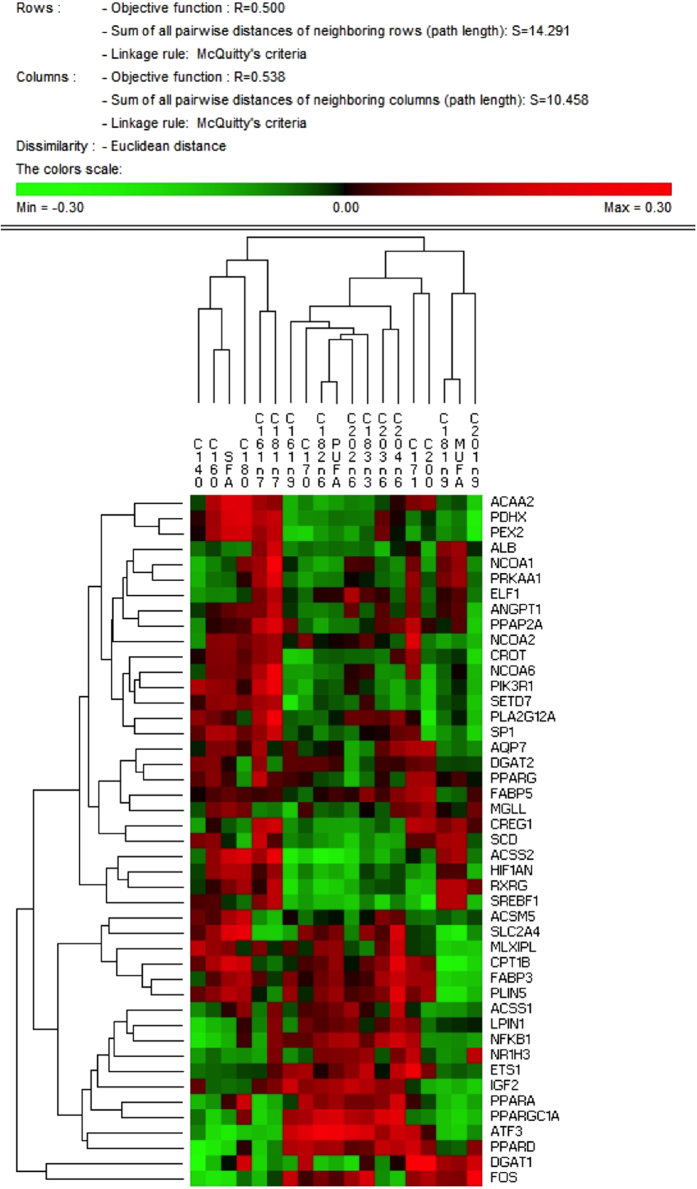
Hierarchical cluster of correlations among gene-expression levels (RQ) of the 45 genes and fatty acid content in muscle.

A significant sex effect (p-value ≤ 0.05) between gene-expression levels was detected in 20 out of the 45 genes analysed (44%): *ACSS1, ACSS2, ATF3, CREG1, DGAT2, ETS1, FABP5, HIF1AN, IGF2, NCOA2, NCOA6, PLA2G12A, PPARA, PPARG, PPARGC1A, PRKAA1, PEX2, SCD, SP1 and SREBF1* (Fig. 1). Interestingly, some of the sex-biased genes identified are key regulators of lipid metabolism, such as *PPARA, PPARG, PPARGC1A* and SREBF1. Note that within the sex-biased genes, several lipogenic genes were more expressed in females (*DGAT2, NCOA2, NCOA6, PPARG, PRKAA1, SCD, SP1 and SREBF1*), whereas more lipolytic genes were over-expressed in males (*ATF3, PPARA and PPARGC1A*). Sexually dimorphic gene expression of genes involved in lipid metabolism has already been described in muscle as well as in other tissues such as liver^19^,^20^. Consistent with our results, *PPARA and SREBF1* presented sex-biased gene expression in human skeletal muscle^21^. Furthermore, *PPARGC1A and PPARA* gene expression in muscle are affected under diet supplementation with 17β-estradiol (E_2_)^21^. In addition, the *SCD and PPARG* mRNA expressions have been observed to be lower in male than in female human muscle cell cultures^22^. To assess the relationship between muscle gene-expression levels and phenotypes, a hierarchical cluster analysis of the correlation values between the gene expression levels of the 45 genes and the fatty acid content in muscle was performed (Fig. 2). The hierarchical cluster analysis showed that genes mainly related to lipogenic pathways (*DGAT2, PPARG, SCD, MGLL, NCOA1, NCOA2, NCOA6, PRKAA1, SP1*, *SREBF1*) clustered together, whereas genes mainly related to lipolytic pathways (*ATF3, CPT1B, PPARA, PPARD, PPARGC1A*) joined together (Fig. 2). Genes within the lipogenic-related cluster showed, in general, positive correlations with palmitoleic (C16:1(n-7)) and octadecenoic (C18:1(n-7)) FAs, while the lipolytic-related group showed a positive correlation with PUFAs in general, specifically with the linoleic (C18:2(n-6)) FA. Overall, these results are in agreement with a previous muscle RNA-Seq transcriptome study of animals which were extreme for IMFA composition performed by our group^16^, where a higher expression of genes related to lipogenic pathways was observed in the muscle of animals with high MUFA and SFA content in muscle.

The highest correlations for gene-expression values (p-value < 1.00 × 10^−16^) were observed for *PPARG* and *SCD* (r = 0.78), *DGAT2* and *PPARG* (0.85), *SCD* and *DGAT2* (r = 0.77), *FABP3* and *PLIN5* (r = 0.83), *FABP3* and *AQP7* (r = 0.80), *ACAA2* and *FABP3* (r = 0.78) and *ELF1* and *PPAP2A* (r = 0.75). Interestingly, the strong correlation between *PPARG* and *SCD* mRNA expression (r = 0.78; p-value < 1.00 × 10^−16^) suggests a transcriptional regulation of *SCD* by the PPARG nuclear factor.

Here, *PPARG, DGAT2* and *SCD*, which are involved in the triacylglycerol synthesis, were highly correlated. Similarly, *FABP3*, *AQP7* and *PLIN5*, which encode for proteins responsible for lipid and glucose transport, were also highly correlated. Remarkably, these three genes were over-expressed in animals with higher MUFA and SFA content in muscle, when compared with animals having more PUFA in a RNA-Seq study^16^.

### eQTL identification

**Figure 3:**
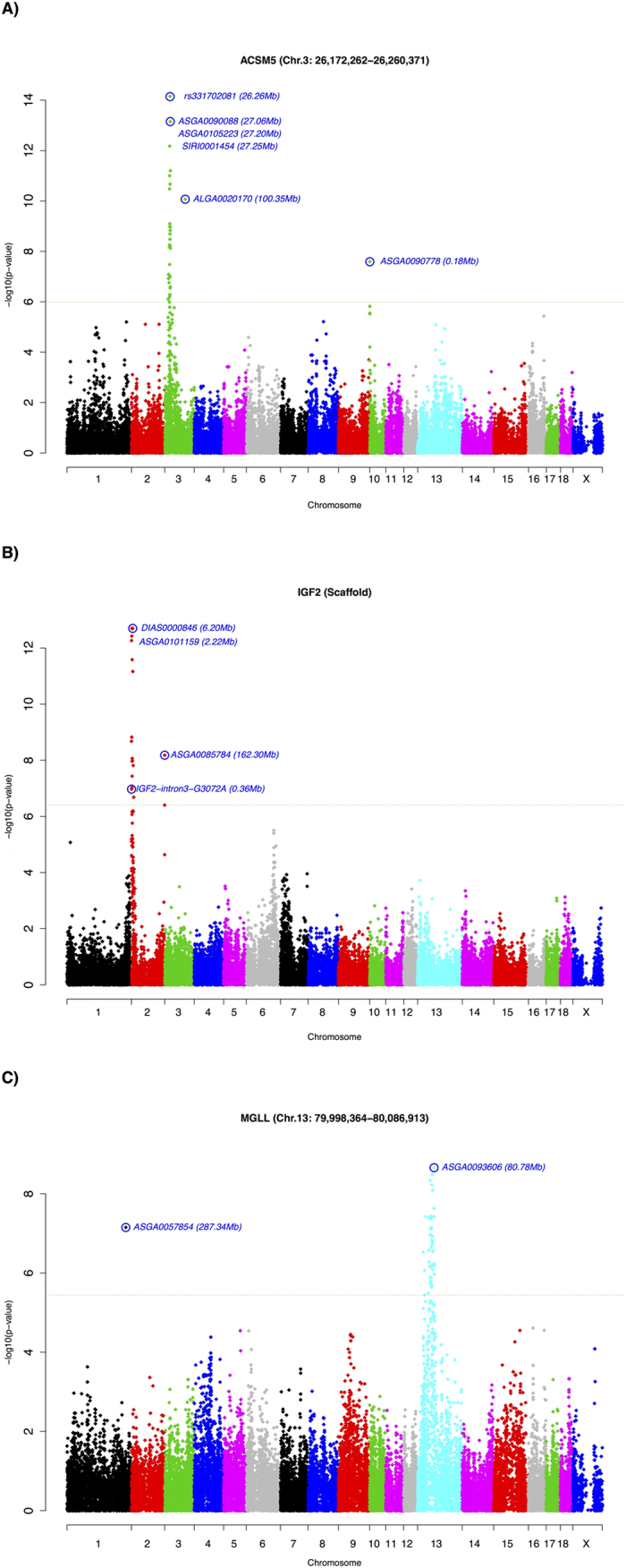
GWAS plot of *ACSM5, IGF2* and *MGLL* gene expression in muscle tissue. Positions in Mb are relative to *Sscrofa10.2* assembly of the pig genome. Horizontal dashed lines indicate the genome-wide significance level (FDR-based q-value ≤ 0.001). Plot of eGWAS for (**A**) *ACSM5* gene expression in muscle (**B**) *IGF2* gene expression in muscle (**C**) *MGLL* gene expression in muscle.

An eGWAS was performed using a total of 40,586 SNPs and the mRNA expression values of the 45 lipid-related genes of 114 BC1_LD animals. A total of 241 SNPs located in 18 *Sus scrofa* chromosomal regions of SSC1, SSC2, SSC3, SSC6, SSC8, SSC9, SSC10, SSC11, and SSC13 were identified for 11 genes: *ACSM5, CROT, FABP3, FOS, HIF1AN, PIK3R1, PLA2G12A, MGLL, IGF2, NCOA1* and *PPARA* (FDR < 0.05; Table 1). Five genes (*ACSM5, IGF2, MGLL, PLA2G12A* and *PPARA*) presented more than one associated eQTL (Table 1). Three out of 18 eQTLs were identified as *cis*-acting, for *ACSM5, IGF2* and *MGLL* gene expression (Fig. 3), suggesting the presence of a mutation in the same gene directly affecting its expression, whereas 16 eQTLs had *trans* regulatory effects. The majority of eQTLs (8 of 18) were located on SSC2 and SSC6 (Table 1).

From the associated SNPs (n = 241), 215 SNPs were successfully annotated with the Variant Effect Predictor (VEP) of Ensembl (Sscrofa 10.2 annotation release 80) of which 54% (117 SNPs) were located in intergenic regions. The remaining 46% (98) of SNPs were mapped within 76 genes: 69 (32%) in introns, 10 in the 5′ flanking region, 13 in the 3′ flanking region, three in the 3′UTR region, two in the coding regions of genes and determining synonymous mutations, and one missense mutation (Supplementary Table S2). Twelve out of the 98 intragenic SNPs were located within a gene exerting a lipid metabolism function (*ABCA3, ACSM3, LRP5, LHFPL4, BRPF1, HRH1, PPARG, ACAD9, COPG1, MGLL, ACAD11, ARHGAP26*) (Supplementary Table S2), but only six of them were expressed in muscle (*ACSM3, PPARG, ACAD9, MGLL, ACAD11* and *ARHGAP26*), according to the Gene Expression Atlas^23^ and the RNA-Seq experiment performed in individuals from the BC1_LD^16^ (Supplementary Table S2). Only one of the intragenic SNPs mapping within genes with a lipid-related function was identified in a *trans* eQTL: the *ARHGAP26* gene located in a *trans*-eQTL for the *PPARA* gene. This gene is activated *via* lipid interaction, however, its role has not been well-defined^24^.

#### *cis*-eQTL identified

The *Insulin-Like Growth Factor 2* (*IGF2*) gene is not mapped in the current *Sscrofa10.2* assembly^25^. However, it has been located by linkage map in the telomeric end of the p arm of SSC2^26^. An intronic *IGF2* mutation (*IGF2*-intron3-G3072A) was described by Van Laere *et al*.^27^ to have a major effect on muscle growth in pigs. Although this mutation only segregated in a small family in the IBMAP F_2_ population^28^, the *cis*-eQTL for *IGF2* suggests that the intronic mutation identified by Van Laere *et al*.^27^ may segregate in the IBMAP BC1_LD, having an important effect on the traits analysed. However, after genotyping the *IGF2*-intron3-G3072A mutation in the BC1_LD animals, we observed that this SNP was not the most associated (p-value = 1.07 × 10^−7^) with the *IGF2* mRNA expression levels (Fig. 3B), suggesting that other mutations in the *IGF2* gene may be the responsible for the mRNA variation in the BC1_LD population. Further analysis will be conducted to validate our hypothesis.

Concerning the *Monoglyceride Lipase* (*MGLL*) eGWAS results, one of the annotated *cis*-SNPs (ASGA0103932) was mapped within an intronic region of the *MGLL* gene (Supplementary Table S2). Nevertheless, this SNP was not the most significant, associated SNP (ASGA0103932; p-value = 2.34 × 10^−8^), suggesting the presence of another polymorphism within or near this gene as the causative mutation affecting *MGLL* gene expression levels. The most significant *cis*-SNP for *MGLL* (ASGA0093606; p-value = 2.20 × 10^−9^) was located less than 0.69 Mb downstream of the *MGLL* gene. In addition, an SNP (ISU10000701) in the *MGLL cis*-eQTL was annotated in the upstream region of the *PPARG* gene, more than 4 Mb from the *MGLL* gene (Supplementary Table S2). *PPARG* and *MGLL* were reported to be co-associated in a previous study of our group for growth and fatness traits, where *PPARG* was described as a major regulator^17^. Additionally, the literature-based analysis with Genomatix also identified an interaction between these two genes (Supplementary Fig. S1), and a chromatin immunoprecipitation (ChiP) experiment performed in epithelial cultured cells revealed PPARG binding sites in the distal *MGLL* promoter^29^. Therefore, it is likely that this region, initially considered as a single *cis*-acting eQTL, in fact comprises two eQTLs. The correlation between *PPARG* and *MGLL* gene expression in muscle was moderate (r = 0.48; p-value = 8.28 × 10^−8^), reinforcing the hypothesis that at least two factors may determine the different levels of *MGLL* mRNA. In this regard, the *MGLL* gene expression would be affected by a variant present in the same gene (*MGLL*), and also by *PPARG.* However, both markers (ASGA0093606 and ISU10000701) were in strong LD (R^2^ = 0.99), not being able to assess whether *MGLL* gene expression is affected by *cis*-, *trans*- or both *cis*-/*trans*- factors on SSC13. Further investigation with other populations is required to reveal this. Remarkably, the significantly associated SNP inside the *PPARG* gene (ISU10000701) was one of the three main co-associated SNPs in the network identified in Puig-Oliveras *et al*.^17^ involved in the determination of growth and fatness traits. Nonetheless, no association was found between the SNP within *PPARG* (ISU10000701) and the *PPARG* gene expression.

Finally, three SNPs (ASGA0090088, ASGA0105223 and SIRI0001454) in complete linkage disequilibrium (R^2^ = 1) were the most significant ones in the *cis*-eQTL (p-value = 7.12 × 10^−14^) associated with the mRNA levels of the *Acyl-CoA Synthetase Medium-Chain Family Member 5* (*ACSM5*) (Table 1). The ASGA0090088 marker was the closest *cis*-SNP, mapping at approximately 798 kb from the upstream region of the *ACSM5* gene (Supplementary Table S2). Notably, after amplifying and sequencing the proximal promoter region of the *ACSM5* gene in ten BC1_LD animals, three polymorphisms were identified at positions g26260919C > T (rs323520560), g26260505G > A (rs339587799) and g26260422G > A (rs331702081), according to the Ensembl ENSSSCG00000026453 sequence, which seem to be in linkage disequilibrium. The most proximal 5′ mutation (g26260422G > A) was genotyped in the BC1_LD animals, and the association analysis of this polymorphism with the *ACSM5* mRNA expression values revealed that this SNP was the most associated one (p-value = 7.11 × 10^−15^; Fig. 3A). Further analyses are necessary to determine the role of these polymorphisms in the transcriptional regulation of the *ACSM5* gene.

#### *trans*-eQTL identified

Table 2 summarizes all the relevant lipid-related genes mapped in the *trans*-eQTL regions associated with the genes analysed.

*ACSM5* gene expression was also associated in *trans* with two chromosomal regions on SSC3 and SSC10. The most associated SNP on SSC10 was an intronic polymorphism (ASGA0090778; p-value = 2.55 × 10^−8^) in the *Component of Oligomeric Golgi Complex 7* (*COG7*) gene (Supplementary Table S2). Although the *COG7* gene has not been described to have a direct relationship with lipid metabolism function, it is reported that members of the COG complex are involved in intra-golgi trafficking and glycosylation of proteins and lipids^30^. Other lipid-related genes were identified within this *trans*-eQTL on SSC10 such as the *NADH Dehydrogenase* (*Ubiquinone*) *1, Alpha/Beta Subcomplex, 1, 8 kDa* (*NDUFAB1*) gene and the *Golgi-associated, Gamma Adaptin Ear Containing, ARF Binding Protein 2* (*GGA2*) gene (Supplementary Table S3). Within the second *trans*-eQTL for *ACSM5* at 100.35 Mb on SSC3, two genes that may affect lipid metabolism were identified: *Protein Kinase C, Epsilon* (*PRKCE*) and *Calmodulin 1* (*Phosphorylase Kinase, Delta*) (*CALM1*) (Supplementary Table S3).

For *FBJ Murine Osteosarcoma Viral Oncogene Homolog* (*FOS*) gene expression, three lipid-related genes in a SSC11 trans-eQTL were identified: *StAR-Related Lipid Transfer (START) Domain Containing 13* (*STARD13*), *Spastic Paraplegia 20 (Troyer Syndrome*) (*SPG20*), and *Arachidonate 5-Lipoxygenase-Activating Protein* (*ALOX5AP*) (Supplementary Table S3). The StAR gene family encodes for globular proteins that form cavities where lipids and lipid hormones bind to be exchanged between biological membranes^31^. Supporting STARD13 as a regulator of *FOS* gene expression, transgenic mice with pancreas *STARD13* ablation showed no detectable mRNA expression of the *FOS* gene^32^.

Two regions on SSC2 and SSC6 were associated in *trans* with the *Peroxisome Proliferator-Activated Receptor Alpha* (*PPARA*) gene expression. In SSC2, several genes related to lipid metabolism were identified: *Rho GTPase Activating Protein 26* (*ARHGAP26*)*, Fibroblast Growth Factor 1* (*Acidic*) (*FGF1*) and *Nuclear Receptor Subfamily 3, Group C, Member 1* (*glucocorticoid receptor*) (*NR3C1*) (Supplementary Table S3). *ARHGAP26* is activated *via* lipid interaction^24^ and may play a role in adipogenesis, as well as the transcription factor *NR3C1*, which maps at approximately 0.5 Mb of the *ARHGAP26* gene. The *FGF1* gene has been identified as being differentially expressed in animals phenotypically extreme for FA composition in muscle^16^. Being noteworthy, FGF1 is involved in preadipocyte differentiation and has been suggested to be acting in the PPARG system, however, the mechanisms remain unclear^33^. In accordance with this hypothesis, it is described that FGF1 can be trans-located to the nucleus, exerting a growth regulatory activity^34^. Hence, it would be important to study the FGF1-PPARA relationship. In the SSC6, *PPARA* trans-eQTL mapped the *Palmitoyl-Protein Thioesterase 1* (*PPT1*) gene, *Metallophosphoesterase 1* (*MPPE1*), *Inositol*(*Myo*)*-1*(*or 4*)*-Monophosphatase 2* (*IMPA2*), and *Cell Death-Inducing DFFA-Like Effector A* (*CIDEA*). Interestingly, *CIDEA*-null mice showed a decreased *PPARA, PPARG* and *SREBF1* gene expression and a decreased *de novo* FA synthesis in the liver^35^. Furthermore, the *CIDEA-PPARA* interaction identified in the eQTL analysis was also captured by Genomatix literature-based analysis (Supplementary Fig. S1). In addition, two melanocortin receptor genes (*MC2R* and *MC5R*) mapped in the *PPARA* SSC6 *trans*-eQTL. In adipocyte cells of *MC2R* knockdown mice alterations in fatty acid composition were observed: a reduction in the C16:1/C16:0 and C18:1/C18:0 ratios and an increase in the arachidonic acid content^36^. On the other hand, MC5R has been associated with obesity phenotypes such as body mass index and fat mass in humans^37^.

The *Phosphoinositide-3-Kinase, Regulatory Subunit 1 Alpha* (*PIK3R1*) eGWAS revealed a trans-eQTL on SSC6 at 145.60 Mb, where several interesting genes involved in lipid metabolism were mapped: *Low Density Lipoprotein Receptor-Related Protein 8, Apolipoprotein E Receptor* (*LRP8*), *24-Dehydrocholesterol Reductase (DHCR24), Dab Reelin Signal Transducer Homolog 1* (*DAB1*), *Protein Kinase, AMP-Activated, Alpha 2 Catalytic Subunit* (*PRKAA2*), *Phosphatidic Acid Phosphatase Type 2B* (*PPAP2B*), and *Proprotein Convertase Subtilisin/Kexin Type 9* (*PCSK9*) (Supplementary Table S3). Interestingly, the *PRKAA2* gene identified within the SSC6 trans-eQTL for *PIK3R1* is a catalytic subunit of the AMP-Activated Protein Kinase (AMPK) which is in charge of regulating lipid synthesis by phosphorylating lipid metabolic enzymes such as ACACA, ACACB, ACC, GYS1, HMGCR, HSL and LIPE to inactivate them. Therefore, it acts regulating key enzymes of fatty acid uptake, esterification, lipolysis and oxidation^38^. In the same way, *PRKAA2* knockdown affects Akt activation. Therefore, PIK3R1 and PRKAA2 are both associated with the PI3K-Akt signalling pathway. Supporting these results, recently published studies suggest that AMPK activates Akt via regulating PI3K^39^. These results highlight *PRKAA2* as a strong candidate gene to explain the variation in the mRNA levels of *PIK3R1*.

Four chromosomal regions (two regions on SSC6, one region on SSC8, and one on SSC9) were associated in *trans* with the *Phospholipase A2, Group XIIA* (*PLA2G12A*) gene expression. Specifically, in the SSC8 eQTL, at approximately 128 to 136 Mb, the *Microsomal Triglyceride Transfer Protein* (*MTTP*), the *Hematopoietic Prostaglandin D Synthase* (*HPGDS*), and the *Alcohol Dehydrogenases 4, 5 and 7* (*ADH7, ADH4*, and *ADH5*) genes involved in lipid metabolism (Supplementary Table S3) were mapped. Interestingly, a polymorphism in the porcine *MTTP* gene has been associated with the lipid transfer activity of the MTTP protein and with the fatty acid composition of fat^40^. *ADH5* was the most expressed gene in pig muscle; meanwhile, the other ADH members (*ADH7* and *ADH4*), jointly with *MTTP* and *HPGDS* genes, showed very low expression levels in muscle^16^, which is in concordance with what was reported in human muscle^23^. Two potential regulators of *PLA2G12A* were identified within the *trans*-eQTL at the 79.9 Mb position on SSC6: *Mitochondrial Trans-2-Enoyl-CoA Reductase* (*MECR*) and *Sestrin 2* (*SESN2*) (Supplementary Table S3). Finally, only one lipid-related gene was identified within the second *trans*-eQTL at 9.7 Mb, the *WW Domain Containing Oxidoreductase* (*WWOX*) gene, which has recently been described to play a role in cholesterol homeostasis and triglyceride biosynthesis^41^.

On SSC9, an eQTL at around 117 Mb affected the expression of genes *PLA2G12A* and the *Hypoxia Inducible Factor 1, Alpha Subunit Inhibitor* (*HIF1AN*) (Table 1). Their mRNA expression was highly correlated (r = 0.60; p-value = 1.98 × 10^−12^), suggesting a common element regulating their transcriptional levels. The three most significant SNPs (H3GA0028012, ASGA0044215, ALGA0117195; p-value = 4.48 × 10^−6^) within this eQTL were in complete linkage disequilibrium (R^2^ = 1) (Table 1). In this region *PIK3CG* was mapped, which encodes a *Phosphatidylinositol-4,5-Bisphosphate 3-Kinase* which is known to participate in different functions, including the insulin-signalling pathway and lipid metabolism^42^, and the *DLD* gene which encodes a *Dihydrolipoamide Dehydrogenase* involved in acetyl-coA biosynthesis (Supplementary Table S3). The *PIK3CG* gene, unlike other PI3K family members, is activated by interaction with G-protein-coupled receptors and by silencing this gene the PI3K-Akt signalling pathway is inhibited^43^. Accordingly, studies in cell lines have suggested that *HIF1AN* gene expression is repressed by a mechanism involving PI3K signalling^44^. Altogether, these results suggest that PRKAA2 may regulate the Class I PI3K Regulatory Subunit 1 (PIK3R1), which is able to form a heterodimer with the PIK3CG catalytic subunit, activate the Akt pathway and inhibit *HIF1AN* gene expression.

For *IGF2* gene expression, apart from the SSC2 *cis*-eQTL, a *trans* eQTL located approximately at 162 Mb in the same chromosome was identified, and the *Sirtuin 3* (*SIRT3*) gene mapped in this region (Supplementary Table S3). It has been described that *SIRT3* knockout mice exhibited decreased oxygen consumption and increased oxidative stress in skeletal muscle^45^. Moreover, *SIRT3* knockout mice showed a down-regulation of Akt phosphorylation^45^ (Supplementary Fig. S2). Members of the *SIRT* gene family have been described as being relevant genes controlling lipolysis and promoting fat mobilization in white adipose tissue^46^, and the *SIRT1* member has been identified as one of the most central genes in a liver co-association network for intramuscular FA composition in IBMAP animal material^18^. Recently, the *SIRT3* member, which can be activated by the AMPK protein, has been suggested as playing a major role in obesity-related diseases^47^.

Finally, within the *trans*-eQTLs identified for *CROT, FABP3*, *MGLL*, and *NCOA1* gene expression, no strong candidate gene exerting a known lipid metabolism function could be detected.

### Functional network analysis of genes mapping in eQTLs

For *trans*-eQTLs, all genes located within a ± 1 Mb interval were selected for gene annotation. Conversely, for *cis*-eQTLs, only the candidate gene studied was considered (*ACSM5*, *IGF2*, and *MGLL*) for further analyses. In the 18 eQTLs, a total of 292 protein-coding genes, 13 miRNA, one miscRNA, six pseudogenes, one rRNA, eleven snoRNAs and four snRNAs were annotated (Supplementary Table S4). From the 292 protein-coding genes with Ensembl Gene ID, 256 had at least one human orthologous gene and were submitted to IPA to perform a functional categorization (Supplementary Table S4).

The main networks identified by IPA analysis were: (i) energy production, small molecule biochemistry and drug metabolism (score = 44); (ii) organismal injury and abnormalities, cancer and haematological disease (score = 42); and (iii) connective tissue disorders, inflammatory disease, skeletal and muscular disorders (score = 37) (Supplementary Table S4).

Focusing on the first network comprising energy production, small molecule biochemistry and drug metabolism functions, we identified the Serine/Threonine Kinase Effector (Akt) complex as central in the network (Supplementary Fig. S2). Remarkably, the Akt complex, which is involved in glucose transport and lipogenesis, was also identified in the muscle transcriptome study of 12 BC1_LD animals which were extreme for intramuscular FA composition^16^. In agreement with these results, several genes (*PIK3CG, PPAP2B, PRKAA2, PTPN2*, and *SIRT3*), identified as potential regulators within the eQTLs of the 45 lipid-related genes, are strongly related to the Akt pathway.

### Identification of master regulators

A total of 298 genes, including (1) the 253 genes annotated in the *trans*-eQTL intervals and, (2) the 45 genes studied of the present study were analysed with iRegulon Cytoscape plugin^48^. We observed that the *EP300* gene was the most enriched transcription factor motif (enrichment score threshold for the motif, NES = 4.737). What was noteworthy was the *EP300* gene was identified as a key regulator of FA composition and IMF traits in the same material using a gene co-association network^18^. On the other hand, five of the 45 genes studied were identified as strong regulators of several genes analysed with iRegulon: *NR1H3* (NES = 5.348; 10 target genes), *MLXIPL* (NES = 4.988; 29 target genes), *PPARA* (NES = 4.785; 32 target genes), *NFKB1* (NES = 3.855; 6 target genes) and *PPARG* (NES = 3.536; 10 target genes). The PPARA motif was present in the highest number of target genes (32 out of 45 genes). Interestingly, the *NR3C1* gene identified within the *PPARA trans*-eQTL on SSC2 was also identified with iRegulon among the regulators for the 45 genes analysed and MatInspector (Genomatix software) predicted a binding site for this transcription factor in the promoter of *PPARA*. The *NR3C1* gene may play a negative role in adipogenesis, regulating the lipolytic and anti-lipogenic gene expression. In human studies, polymorphisms in the *NR3C1* gene have been suggested as contributing to obesity^49^. Afterwards, by using the Genomatix interface, it was observed that NR3C1 interacts with *NFKB*, *CREB, NCOA1* and *NCOA2* genes, and may be affected by the Corticotropin-Releasing Hormone (CRH) and Insulin (INS) (Supplementary Fig. S3). Accordingly, CRH was identified in both network analysis for fatness and growth traits^17^ and the insulin-signalling pathway in a RNA-Seq study comparing animals extreme for their intramuscular FA composition^16^, both studies performed with IBMAP animal material. Thus, we hypothesize that *NR3C1* may be a master regulator of lipid metabolism through the regulation of *PPARA* gene expression. However, further studies are necessary to corroborate this hypothesis.

### QTL and eQTL co-localization

Transcriptomic data can be used to identify candidate genes underlying QTLs through the co-localization with eQTLs. Thus, our group looked for the overlapping of the 241 SNPs with the QTLs described in PigQTLDB^50^. A total of 234 SNPs (97%) co-localized in 132 QTLs for fatness traits and 157 SNPs (65%) within 10 different QTLs for FA composition (Supplementary Table S5), confirming a high co-localization of eQTLs and fat-related QTLs. The large number of QTLs described for these traits, covering a large extension of the porcine genome, provides evidence for a complex genetic pleiotropic regulation basis of these traits.

Notably, five genes identified within the eQTLs (i.e. *ARHGAP6, IGF2, MC2R, MGLL, NR3C1*) overlapped with QTLs described in the IBMAP population. For instance, the *IGF2* gene, for which a *cis*-eQTL was detected, was identified within a confidence interval of one of the epistatic regions affecting muscle fibre traits^51^. The *MGLL* gene, showing a *cis*-acting eQTL, was located close to a GWAS interval affecting FA composition traits: palmitic (C16:0) and oleic (C18:1(n-9)) FAs, the polyunsaturated/saturated ratio (PUFA/SFA ratio), SFA, and unsaturated index (UI)^12^. Furthermore, *MGLL* maps within a QTL interval affecting growth^11^. The *MC2R* gene, identified in a *PPARA trans*-eQTL, maps within an IBMAP QTL region for IMF content and backfat thickness^52^. Moreover, *ARHGAP6* and *NR3C1* genes also identified in a *trans*-eQTL for *PPARA* gene expression are located within a QTL for growth traits in the IBMAP population^11^.

## Conclusions

In the present study, we provide a list of potential candidate genes and genetic variants that may be regulating the transcriptional level of relevant lipid-related metabolism genes. Combined assessment of the results obtained from eGWAS and GWAS may provide complementary information of genes and variants determining the IMF and FA composition traits. Therefore, the present results identify potentially key genes and variants affecting pork-meat quality. However, more efforts should be made to validate our results, for instance, the involvement of the *NR3C1* gene as a major regulator in muscle FA metabolism.

## Methods

### Animal samples and phenotypes

The IBMAP population was obtained by crossing three Guadyerbas Iberian boars with 31 Landrace sows^6^. In the present study, 114 animals were used (65 females and 49 males) belonging to the BC1_LD generation of the IBMAP population obtained by crossing five F1 boars with 26 Landrace sows. Animals were fed *ad libitum* with a cereal-based commercial diet and slaughtered at 179.8 days ±2.6 days. Animal care and procedures were performed following national and institutional guidelines for the Good Experimental Practices and approved by the Ethical Committee of the Institution (IRTA- Institut de Recerca i Tecnologia Agroalimentàries). Samples of the *Longissimus dorsi* muscle were collected, snap-frozen in liquid nitrogen and stored at −80 °C until further RNA isolation. Genomic DNA was extracted from blood samples according to the phenol-chloroform method^53^.

### Selection of lipid-related metabolism genes in muscle

In previous studies of our group, strong candidate genes affecting IMFA content and FA composition of the *Longissimus dorsi* muscle of the IBMAP BC1_LD, 25% Iberian and 75% Landrace, were identified by using GWAS, RNA-Seq and co-association network approaches^12^,^16^,^17^,^18^. In the present study, a list of 45 genes functionally related with lipid metabolism was selected, prioritizing candidate genes for FA composition (Supplementary Table S6).

We included: (1) candidate genes differentially expressed (*ACAA2, AQP7, ALB, ANGPT1, ATF3, MLXIPL, FOS, HIF1AN, PIK3R1, PLIN5, PPARG, SCD, SLC2A4*) in the *Longissimus dorsi* muscle of two phenotypically extreme groups of animals for intramuscular FA composition from the IBMAP cross and their potential regulators (*NFKB1, PPARGC1A*)^16^; (2) candidate functional and positional genes (*FABP5, PIK3R1, PLA2G12A, PPAP2A*) identified in a GWAS study for intramuscular FA composition in the same animal material^12^; and (3) genes related to lipid metabolism identified in gene co-association networks for FA composition^18^ (*ACSM5, ANGPT1, FABP3, FABP5, MGLL, NCOA2, PEX2, PPARG, SETD7*) and fatness and growth- related traits^17^ (*ALB, CREG1, ELF1, FABP5, MGLL, PPARG*). Finally, in order to complete the set of genes, we chose genes which have been described in the literature to play different roles (fatty acid synthesis, transport, storage and oxidation) in muscle lipid metabolism, prioritizing those genes which are expressed in muscle, such as transcriptional factors, co-factors and nuclear receptors (*ETS1, LPIN1, NR1H3, NCOA1, NCOA6, PPARA, PPARD, PRKAA1, RXRG, SP1, SREBF1*)^54^ (freely available at http://www.bioguo.org/AnimalTFDB/), enzymes (*ACSS1, ACSS2, CPT1B, CROT, DGAT1, DGAT2*, *PDHX*), as well as the *IGF2* gene, which has been described as the causal factor of the imprinted QTL for muscle growth and fat deposition in a Meishan × Large White intercross^27^ (Supplementary Table S6). Moreover, polymorphisms in the *IGF2* gene have been significantly associated with muscle FA composition in swine^55^.

### Genotyping

A total of 197 animals from the BC1_LD (160 backcrossed individuals and their respective parents) were genotyped with the Porcine *SNP60 Beadchip* (Illumina), following the Infinium HD Assay Ultra protocol (Illumina)^56^. Raw data were visualized with GenomeStudio software (Illumina) and trimmed for high genotyping quality (call rate >0.99). Markers with minor allele frequency (MAF) >5% and animals with missing genotypes <5% were retained. After the quality control filter, a subset of 40,586 SNPs and 197 animals remained.

Furthermore, the BC1_LD animals were genotyped with two SNPs within the *ACSM5* (rs331702081) and *IGF2* (*IGF2*-intron3-G3072A) genes using Taqman OpenArray^TM^ genotyping plates custom-designed in a QuantStudio^TM^ 12K flex Real-Time PCR System (ThermoFisher Scientific) (Supplementary Table S7) and the pyrosequencing protocol previously described^27^, respectively.

### Gene-expression profiling

Total RNA was isolated from the *Longissimus dorsi* muscle of 114 samples with RiboPure kit (Ambion; Austin, TX, USA). Total RNA was quantified in a NanoDrop ND-1000 spectrophotometer (NanoDrop Products; Wilmington, DE, USA). The RNA was converted to cDNA using the *High-Capacity cDNA Reverse Transcription* (Applied Biosystems). The cDNA samples were loaded into a *Dynamic Array* 48.48 chip in a BioMark system (Fluidigm; San Francisco, CA, USA) through a NanoFlex controller following a protocol previously described^18^. For this experiment, the expressed levels of 48 genes were analysed, and a total of 45 target genes were normalized using the two most stable housekeeping genes (*ACTB* and *TBP*). Primers used for the analyses are detailed in Supplementary Table S8. Data were collected using the Fluidigm Real-Time PCR analysis software 3.0.2 (Fluidigm) and analysed using the DAG expression software 1.0.4.11^57^, applying the relative standard curve method (See Applied Biosystems user bulletin #2). Analyses were performed using the normalized gene-expression levels of each sample and assay. The animals showing abnormal gene expression levels (outliers) were removed and data obtained were normalized if necessary by performing log_2_ transformation of the Normalized Quantity (NQ) value. The sex effect was also tested by using a linear model with R programme^58^.

### Gene-expression association analysis

An eGWAS was also performed using the genotypes of BC1_LD animals and the expression values from muscle. A mixed model was employed implemented on Qxpak 5.0^59^:


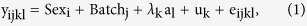


in which y_ijkl_ was the k^th^ individual record, sex (two levels) and batch (five levels) were fixed effects, λ_k_ was a −1, 0, +1 indicator variable depending on the k^th^ individual genotype for the l^th^ SNP, a_l_ represents the additive effect associated with the l^th^ SNP, u_k_ is the infinitesimal genetic effect treated as random and distributed as N(**0**, **A**σ_u_^2^), where **A** is the numerator of the pedigree-based relationship matrix and e_ijkl_ the residual.

The same mixed model was applied to perform the association analyses with the *ACSM5* (rs331702081) and *IGF2* (IGF2-intron3-G3072A) polymorphisms and the *ACSM5* and *IGF2* mRNA expressions, respectively.

To correct for multiple testing, the false discovery rate (FDR) was calculated with the q-value library of the R package setting the threshold at q-value ≤ 0.05^58^,^60^. Given that *cis*-acting eQTLs tend to have larger magnitudes of effect on gene expression than do *trans*-acting eQTLs^61^, and several SNPs are affected by strong LD, a more restrictive threshold to avoid false positives was applied at p-value ≤ 0.001 for those eGWAS showing a *cis*-eQTL (see Q-Q plot in Supplementary Fig. S4).

The SNPs identified were classified as *cis* when they were located within 1 Mb from the gene analysed and as *trans* when they were located elsewhere in the genome. The number of significant SNPs belonging to the same interval was considered among associated SNPs less than 10 Mb apart.

### Gene annotation and functional analysis

The significantly associated SNPs were mapped in the Sscrofa10.2 assembly and were annotated with the Ensembl Genes 80 Database using VEP software^62^. The genomic eQTL intervals considering ±1 Mb around the candidate chromosomal regions were annotated using BioMart software^63^.

The Core Analysis function included in the Ingenuity Pathway Analysis software (IPA 4.0, Ingenuity Systems Inc., http://www.ingenuity.com; Accessed 8 July 2015) and the Genomatix Pathway System (GePS) (Release 2.8.0) in the Genomatix software suite (Genomatix Software GmbH; Munich, Germany; available at: http://www.genomatix.de; accessed 8 October 2015) were used to perform the functional analysis of genes mapped in the 18 eQTLs regions. Specifically, the IPA software was used for data interpretation in the context of biological processes, pathways, networks and upstream regulators. All information generated in this software is derived from the Ingenuity Pathway Knowledge Base (IPKB), which is based on functions and interactions of genes published in the literature. Genomatix was used to retrieve additional information of gene functions, interactions and upstream regulators based on the literature. Furthermore, information from Mouse Genome Database^64^ and Genecards^65^ was used to identify gene functions affecting the phenotypes analysed. For the lipid-related genes, the Gene Expression Atlas^23^ was used to determine whether they were expressed in muscle or not.

Finally, an *in-silico* identification of transcription-factor binding sites in the promoter region of the 256 annotated genes was performed. For this analysis, iRegulon^48^ was used, which relies on the analysis of the motif enrichment for a transcription factor in the gene set using databases of nearly 10,000 TF motifs and 1,000 ChIP-seq data sets or “tracks”.

### Correlation of gene expression and phenotypes

Correlations were performed among gene expression of the 45 genes to explore the relationship between genes. Furthermore, pairwise correlations among gene expression and FA composition percentages in muscle^12^ were carried out to explore the relationships between gene expression and phenotypes. All values were normalized applying the log_2_ of raw data if necessary. Afterwards, gene expression was corrected by sex (two levels) and batch (five levels) effects, whereas FA composition percentages were corrected by sex, batch and carcass weight. The remaining residuals of the phenotypes and gene-expression values corrected for the corresponding effects were used to obtain the pairwise correlations. The hierarchical clustering option of PermutMatrix software^66^ was used to visualize the results of both traits and genes.

## Additional Information

**How to cite this article**: Puig-Oliveras, A. *et al*. Expression-based GWAS identifies variants, gene interactions and key regulators affecting intramuscular fatty acid content and composition in porcine meat. *Sci. Rep.* **6**, 31803; doi: 10.1038/srep31803 (2016).

## Supplementary Material

Supplementary Information

Supplementary Table S2

Supplementary Table S3

Supplementary Table S5

## Figures and Tables

**Table 1:** Significant eQTLs identified.

No.	Gene	Most significant SNPs in eQTL	p-value	q-value	eQTL interval^a^	No. SNPs^b^	Type of eQTL
1	*ACSM5*	ASGA0090088, ASGA0105223, SIRI0001454	7.12E-14	9.29E-10	3:16487448-27941321	43	*CIS*
2	*ACSM5*	ALGA0020170	8.58E-11	3.73E-07	3:100347076	1	*TRANS*
3	*ACSM5*	ASGA0090778	2.55E-08	3.44E-05	10:175359	1	*TRANS*
4	*CROT*	ALGA0046590	4.01E-07	9.77E-03	8:15870120-15912410	3	*TRANS*
5	*FABP3*	ALGA0063896	1.26E-06	4.47E-02	11:81364018-82160789	2	*TRANS*
6	*FOS*	H3GA0031293	1.22E-09	4.95E-05	11:6736568-11968518	6	*TRANS*
7	*HIF1AN, PLA2G12A*	H3GA0028012, ASGA0044215, ALGA0117195	2.91E-07	3.92E-03	9:117742788-117851340	3	*TRANS*
			4.48E-06	2.74E-02			
8	*IGF2*	ASGA0101159, DIAS0000846	<1.0E-25	<1.0E-25	2:16416-11175095	17	CIS*
9	*IGF2*	ASGA0085784	6.63E-09	2.45E-05	2:162088043-162298086	2	*TRANS*
10	*MGLL*	ASGA0057854	7.06E-08	6.28E-05	1:287348708	1	*TRANS*
11	*MGLL*	ASGA0093606	2.20E-09	2.61E-05	13:27954256-82589660	147	*CIS*
12	*NCOA1*	ALGA0016576, MARC0045025, MARC0087200	2.60E-06	3.49E-02	2:146195530-146722998	3	*TRANS*
13	*PIK3R1*	ALGA0117336	1.24E-06	3.02E-02	6:143952619-145605378	3	*TRANS*
14	*PLA2G12A*	ALGA0113789	1.96E-08	7.84E-04	6:10020702	1	*TRANS*
15	*PLA2G12A*	ALGA0103867	3.41E-06	2.74E-02	6:79924228	1	*TRANS*
16	*PLA2G12A*	ASGA0039774	1.34E-06	2.68E-02	8:128899782-136113975	3	*TRANS*
17	*PPARA*	M1GA0003328	3.25E-06	3.30E-02	2:150634202	1	*TRANS*
18	*PPARA*	MARC0074986, DIAS0004325, CASI0006620	6.17E-07	6.17E-07	6:89986075-90352248	3	*TRANS*

^a^Chromosomal location is given according to the Sscrofa10.2 assembly coordinates. Positions are relative to the significant eQTL interval start and end. Lengths are given in base-pairs.

^b^Number of significant SNPs within the eQTL interval.

^*^Approximate location of the gene described by Fontanesi *et al*.^26^.

**Table 2:** *Trans*-eQTL annotation.

Gene	Chromosome	Candidate genes within the eQTL
*ACSM5*	SSC3	*CALM1, PRKCE*
	SSC10	*COG7, GGA2, NDUFAB1*
*FOS*	SSC11	*ALOX5AP, SPG20, STARD13*
*PPARA*	SSC2	*ARHGAP26, FGF1, NR3C1*
	SSC6	*CIDEA, IMPA2, MC2R, MC5R, MPPE1, PPT*
*PIK3R1*	SSC6	*DAB1, DHCR24, LRP8, PCSK9, PPAP2B, PRKAA2*
*PLA2G12A*	SSC6	*MECR, SESN2, WWOX*
	SSC8	*ADH4, ADH5, ADH7, HPDGS, MTTP*
	SSC9	*PIK3CG, DLD*
*IGF2*	SSC2	*SIRT3*
