# Author Correction: Expression-based GWAS identifies variants, gene interactions and key regulators affecting intramuscular fatty acid content and composition in porcine meat

**DOI:** 10.1038/s41598-022-08878-7

**Published:** 2022-03-22

**Authors:** Anna Puig-Oliveras, Manuel Revilla, Anna Castelló, Ana I. Fernández, Josep M. Folch, Maria Ballester

**Affiliations:** 1 Departament de Ciència Animal i dels Aliments, Universitat Autònoma de Barcelona (UAB), 08193 Bellaterra, Spain; 2 Plant and Animal Genomics, Centre de Recerca en Agrigenòmica (CRAG), 08193 Bellaterra, Spain; 3 Departamento de Genética Animal, Instituto Nacional de Investigación y Tecnología Agraria y Alimentaria (INIA), 28040 Madrid, Spain; 4 Departament de Genètica i Millora Animal, Institut de Recerca i Tecnologia Agroalimentàries (IRTA), Torre Marimon, 08140 Caldes de Montbui, Spain

Correction to: *Scientific Reports* 10.1038/srep31803, published online 18 August 2016

The Acknowledgments section in the original version of this Article is incomplete.

 “The authors gratefully acknowledge J.L. Noguera (Institut de Recerca i Tecnologia Agroalimentàries; IRTA) for the animal material contribution. This work was funded by the Ministerio de Economía y Competitividad (MINECO AGL2011-29821-C02 and AGL2014-56369-C2). A. Puig-Oliveras was funded by a “Personal Investigador en Formació” (PIF) grant from Universitat Autònoma de Barcelona (458-01-1/2011). M. Revilla was funded by a Formació i Contractació de Personal Investigador Novell (FI-DGR) Ph.D Grant from the Generalitat de Catalunya (ECO/1639/2013). M. Ballester is financially supported by a Ramon y Cajal contract (RYC-2013-12573) from the Spanish Ministry of Economy and Competitiveness.”

should read:

“The authors gratefully acknowledge J.L. Noguera (Institut de Recerca i Tecnologia Agroalimentàries; IRTA) for the animal material contribution. This work was funded by the Ministerio de Economía y Competitividad (MINECO AGL2011-29821-C02 and AGL2014-56369-C2) and the Fondo Europeo de Desarrollo Regional (FEDER). A. Puig-Oliveras was funded by a “Personal Investigador en Formació” (PIF) grant from Universitat Autònoma de Barcelona (458-01-1/2011). M. Revilla was funded by a Formació i Contractació de Personal Investigador Novell (FI-DGR) Ph.D Grant from the Generalitat de Catalunya (ECO/1639/2013). M. Ballester is financially supported by a Ramon y Cajal contract (RYC-2013-12573) from the Spanish Ministry of Economy and Competitiveness.”

