# Supplementary material for: Expression-based GWAS identifies variants, gene interactions and key regulators affecting intramuscular fatty acid content and composition in porcine meat
Source: Sci Rep. 2016 Aug 18;6:31803. doi: 10.1038/srep31803 (PMC4989154; doi:10.1038/srep31803)
Supplement: Supplementary Information [file srep31803-s1.pdf]

SUPPLEMENTARY MATERIAL

Expression-based GWAS identifies variants, gene interactions and key regulators affecting intramuscular fatty acid content and composition in porcine meat

Anna Puig-Oliveras[1,2 *], Manuel Revilla[1,2], Anna Castelló[1,2], Ana I. Fernández[3], Josep M. Folch[1,2], Maria Ballester[1,2,4]

[1] Departament de Ciència Animal i dels Aliments, Universitat Autònoma de Barcelona (UAB), 08193 Bellaterra, Spain

[2] Plant and Animal Genomics, Centre de Recerca en Agrigenòmica (CRAG), 08193 Bellaterra, Spain

[3] Departamento de Genética Animal, Instituto Nacional de Investigación y Tecnología Agraria y Alimentaria (INIA), 28040 Madrid, Spain

[4] Departament de Genètica i Millora Animal, Institut de Recerca i Tecnologia Agroalimentàries (IRTA), Torre Marimon, 08140 Caldes de Montbui, Spain.

*E-mail: anna.puig.oliveras@hotmail.com

Figure S1. Network of the lipid metabolic process function obtained by Genomatix.

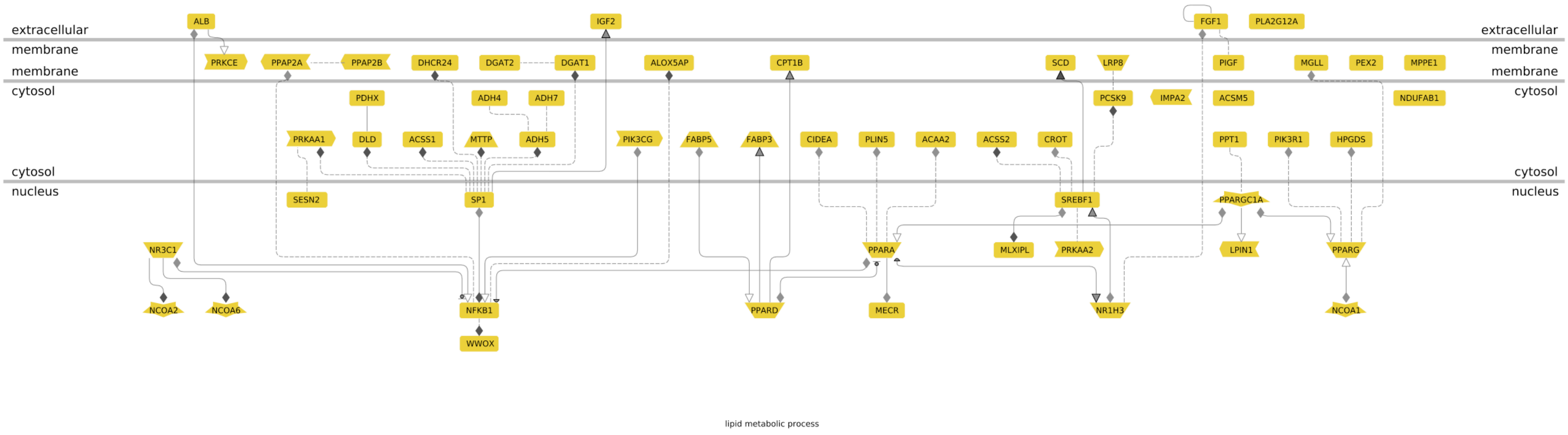

Supplementary Figure S2. Network (score 44) generated by IPA of 25 focus genes corresponding to the energy production, small molecule biochemistry and drug metabolism functions.

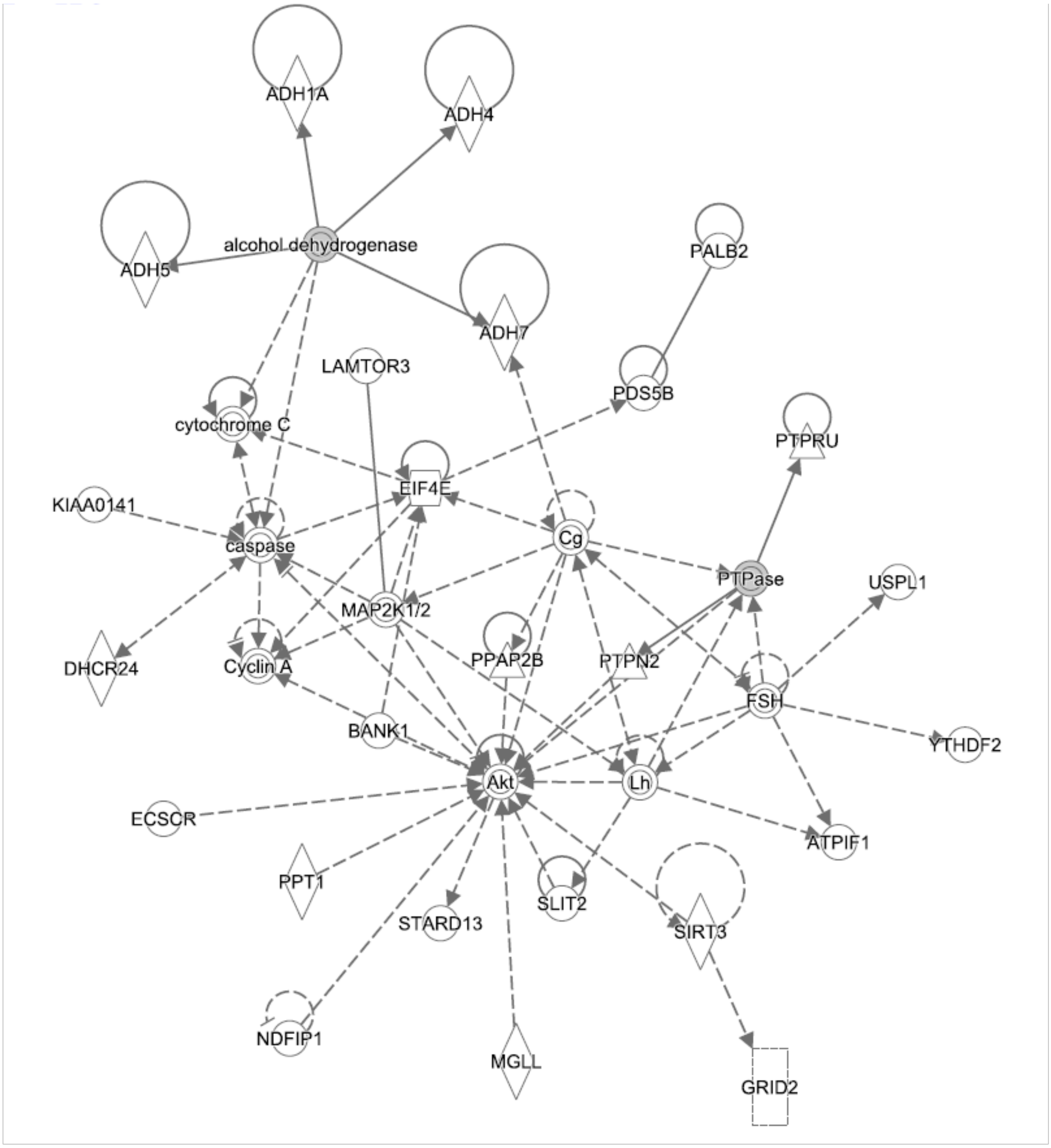

Figure S3. Literature-based network of genes interacting with NR3C1 transcription factor generated by Genomatix.

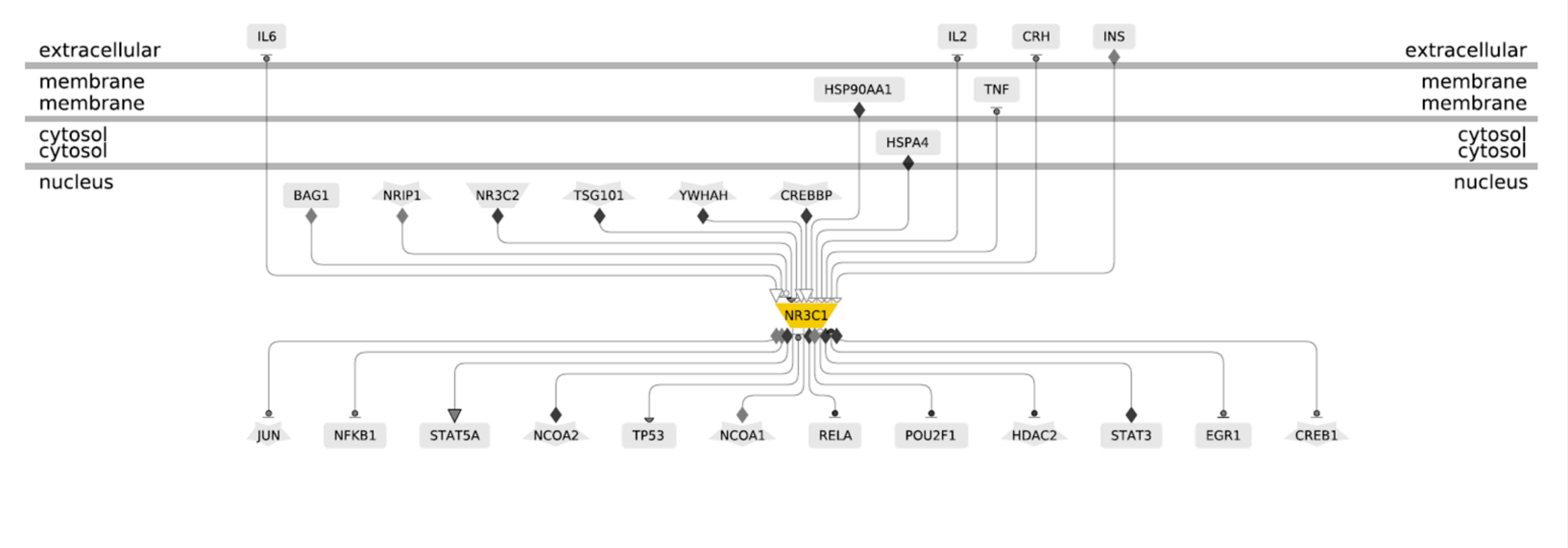

Figure S4. Q-Q plot representing the *P*-value distribution of the *ACSM5, IGF2* and *MGLL* gene eGWAS. The expected distribution of the *P*-values is indicated with a red line, whereas black points represent the observed distribution. Q-Q plot of p-value distribution for (A) *ACSM5* gene expression in muscle (B) *IGF2* gene expression in muscle (C) *MGLL* gene expression in muscle.

A)

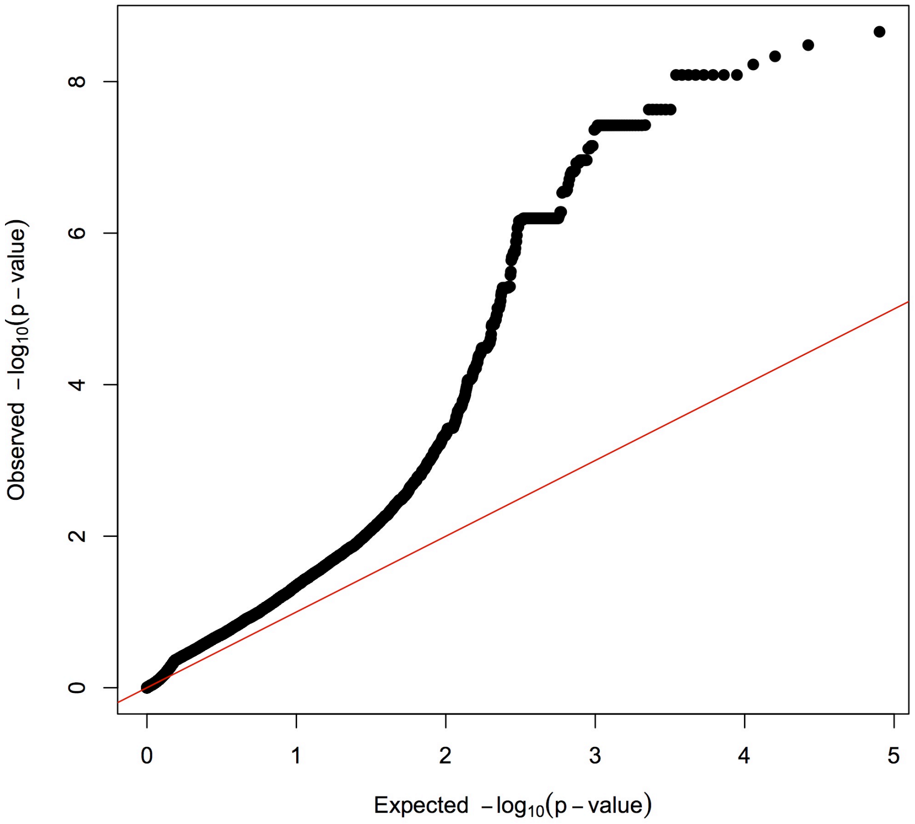

B)

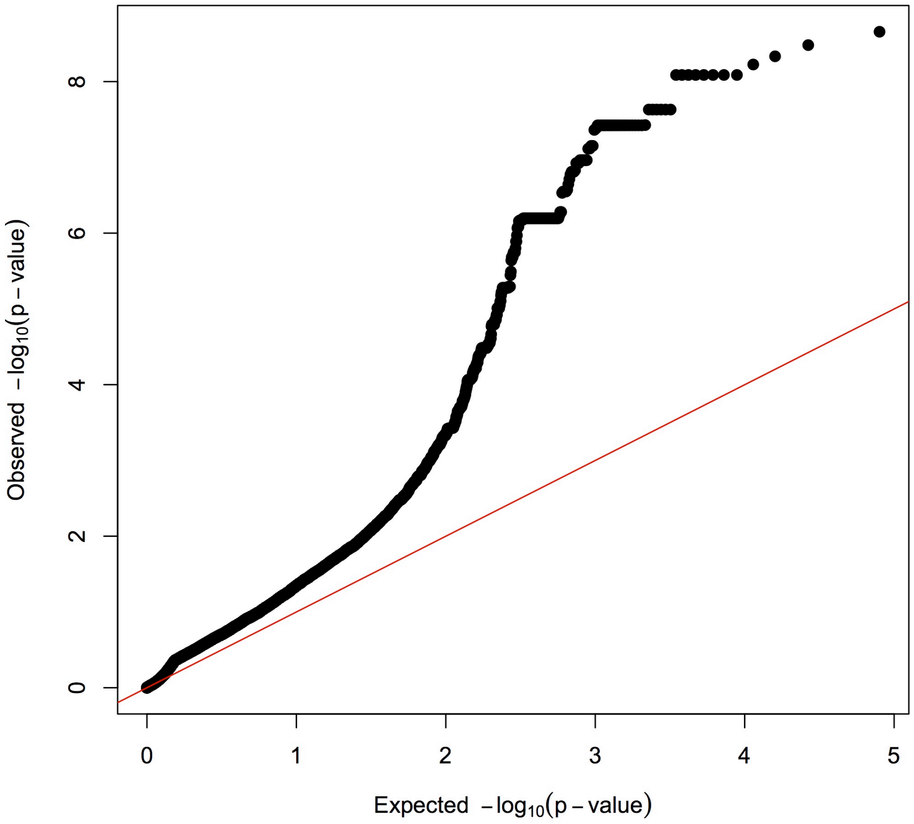
Observed $-\log_{10}(p-\text{value})$
Expected $-\log_{10}(p-\text{value})$
0
2
4
6
8
0
1
2
3
4
5

C)

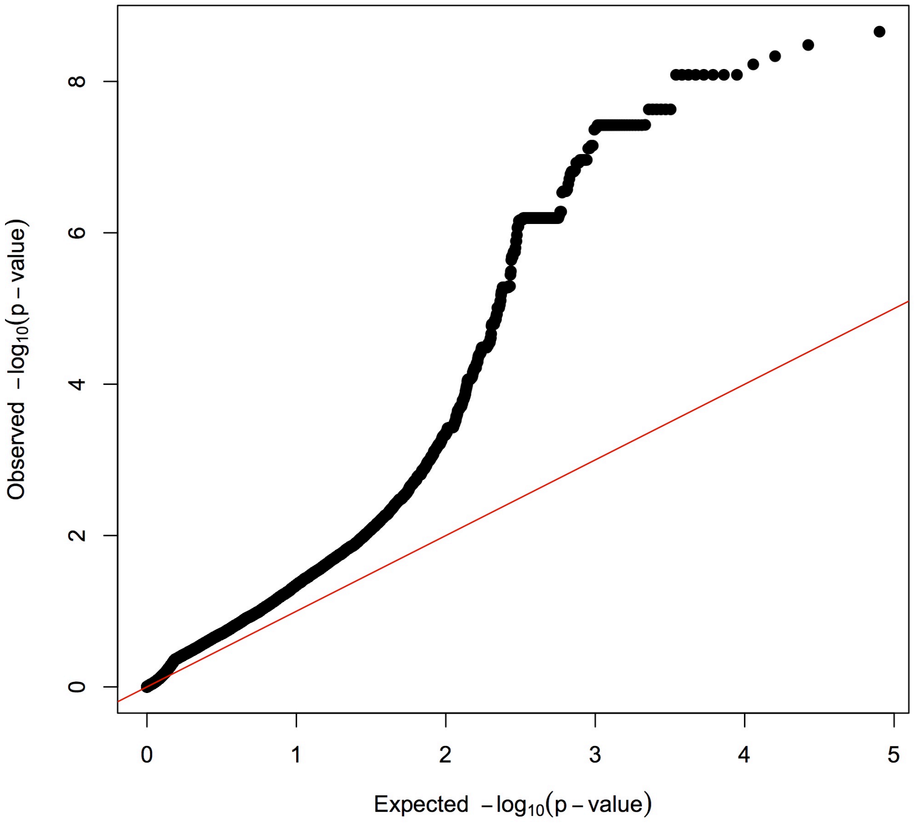
Observed $-\log_{10}(p-\text{value})$
Expected $-\log_{10}(p-\text{value})$
0
2
4
6
8
0
1
2
3
4
5

Table S1. Descriptive statistics including mean and standard deviation (SD) of intramuscular fat (IMF), fatty acid (FA) composition and fatty acid indices of the BC1_LD animals analyzed. *The percentage of each FA, relative to the total FA.

Carcass quality	Abbreviation	Mean	SD
**Meat quality** (n=98)			
Intramuscular fat (%)	IMF	2.10	0.07
**Fatty acids** (n=114)			
Myristic acid	C14:0	1.18	0.01
Palmitic acid	C16:0	22.55	0.12
Heptadecenoic acid	C17:0	0.26	0.01
Stearic acid	C18:0	14.19	0.09
Arachidic acid	C20:0	0.26	0.01
Saturated FA	SFA	38.43	0.16
Palmitoleic acid	C16:1(n-7)	2.49	0.04
cis-7 hexadecenoic acid	C16:1(n-9)	0.39	0.01
Heptadecenoic acid	C17:1	0.26	0.01
Oleic acid	C18:1(n-9)	40.09	0.28
Octadecenoic acid	C18:1(n-7)	3.88	0.03
Eicosenoic acid	C20:1(n-9)	0.85	0.01
Monounsaturated FA	MUFA	47.96	0.30
Linoleic acid	C18:2(n-6)	10.38	0.24
α-Linolenic acid	C18:3(n-3)	0.66	0.03
Eicosadienoic acid	C20:2(n-6)	0.54	0.01
Dihomo-gamma-linolenic acid	C20:3(n-6)	0.27	0.01
Arachidonic acid	C20:4(n-6)	1.54	0.07
Polyunsaturated FA	PUFA	13.39	0.33

Table S4. Top functional networks identified with IPA based on the list of annotated genes mapping within the 18 eQTLs.

ID	Top Diseases and Functions	Score	Focus Molecules	Molecules in Network
1	Energy Production, Small Molecule Biochemistry, Drug Metabolism	44	25	*ADH4,ADH5,ADH7,ADH1A,Akt,alcohol dehydrogenase,ATPIF1,BANK1, caspase,Cg, Cyclin A,cytochrome C,DHCR24,ECSCR,EIF4E,FSH,GRID2,KIAA0141,LAMTOR3, Lh,MAP2K1/2,MGLL,NDFIP1,PALB2,PDS5B,PPAP2B,PPT1,PTPase,PTPN2,PTPRU, SIRT3,SLIT2,STARD13,USPL1,YTHDF2*
2	Organismal Injury and Abnormalities, Cancer, Hematological Disease	42	24	*Dynein,EFNB2,EMCN,ENaC,EPB41,ERK1/2,Fgfr,IFITM3,Ige,KL,LDL-cholesterol, MED18,mediator,MHC Class II (complex),MTTP,NAMPT,NRG2,PCSK9,PIGF,PLC gamma,PSMD13,RFXAP,RIC8A,SCNN1B,SLC26A4,Smad1/5/8,SPG20,SPRY4, SUPT20H,TNFRSF8,TNFSF15,TNFSF13B,TUBB6,Ubiquitin,UNC5C*
3	Connective Tissue Disorders, Inflammatory Disease, Skeletal and Muscular Disorders	37	22	*BRD8,BTF3,Cbp/p300,CDC25C,CEP76,CIDEA,Ck2,DNAJB14,DNAJC8,DNAJC18,Gro wth hormone,Histone h4,HMGB1,HSP,Hsp70,Hsp22/Hsp40/Hsp90, HSPA9,HSPB11, HSPH1,IL12 (complex),Interferon alpha,Jnk,KLHL3,NME5, NR3C1,PDLIM5, phosphatase,POMP,PPP3CA,PRKCE,Proinsulin,PSMG2, RHOQ,SESN2,STAT5a/b*
4	Connective Tissue Disorders, Dermatological Diseases and Conditions, Developmental Disorder	26	17	*ALOX5AP,Alp,Ap1,CXXC5,DIO1,FGF1,HDL,IFITM1,Ifn,IFN Beta,IL1,LAMB1,LDL, MAB21L1,Mek,METAP1,NADPH oxidase,NFkB (complex),Nos,Nr1h,PAPPA,Pdgf (complex),PDGF BB,Pro-inflammatory Cytokine,RAP1GDS1,SIGIRR,SIL1,SLC7A1, SMAD9,SOCS5,Tgf beta,Tlr,TMEM173,TSH,ZMPSTE24*
5	Cell-To-Cell Signaling and Interaction, Cellular Assembly and Organization, Cellular Function and Maintenance	26	17	*Actin,ALG5,Alpha Actinin,Alpha catenin,ATOH1,BCR (complex),CAP1,CBLL1,CCNA1, COL9A2,Collagen type I,CTNNA1,Cyclin E,DAPP1,E2f,EGR1,ERK,estrogen receptor,EXOSC8,F Actin,FRY,IGF2,IgG,IgG2a,Igm,Immunoglobulin,MZB1,Nfat (family),NRCAM,p70 S6k,PCDH1,PI3K (complex),SLC26A3,TCF,TNC*
6	Gene Expression, Protein Synthesis, Cancer	24	16	*AFG3L2,APP,ATL3,BET1L,BMPR1B,BSND,CIART,COG5,COG7,CREB1,DDIT4L,FAM 212B,FAM3A,GGA2,HRASLS,IGSF10,KCNC4,MEDAG,MPI,MPV17L,MYOT,NLN,OCIA D2,PACRGL,PKP3,POMZP3,PPARG,SEH1L,SERTM1,TCEANC2,TP53,UBL3,WARS2, ZDHHC23,ZNF35*
7	Nervous System Development and Function, Lipid Metabolism, Nucleic Acid Metabolism	22	15	*ARGLU1,ARTN,AUH,DCTN3,DCTN5,DCTN6,DECR2,EPHA5,GFRA3,IMPA1,IMPA2,IM PAD1,KATNAL1,MCFD2,MECR,MON1B,N4BP2L2,NAA38,NBEA,NDC1,REEP6,RET,R NF19A,SCARA3,SDK1,SRBD1,TMEM59,TSPAN3,TSPAN4,TSPAN5,TSPAN6,TTC4,U BC,VPS16,VPS41*
8	Cellular Development, Cellular Growth and Proliferation, Hematological System Development and Function	22	15	*ARHGAP19,BCAP29,CASC4,CDH9,CTNNB1,DIP2B,DMRTB1,ELAVL1,FAM110B,FAM 13B,FAM53C,GNB1,HHAT,HIF1A,HPGDS,IFITM5,KCNIP4,MPV17L,NEDD9,NUDT7,O DF3,PANK1,PSD2,RLF,SAMD1,SPIRE1,SUMO2,TAF12,TBCB,TGFB1,TMEM19,TTC2 2,Wnt,WNT8A,YWHAQ*
9	Cardiovascular Disease, Hereditary Disorder, Metabolic Disease	20	14	*ANKEF1,ATHL1,ATP6V1E2,C4orf17,C9orf114,CACUL1,CEP192,DENND5B,DENND6 A,DEPDC5,DLD,GLT8D2,GTPase,HNRNPA0,KDM1A,MPPE1,MRPL33,MTO1,NDUFA B1,PAXIP1,RCC1,RGP1,RIC1,RNF14,RPL5,SPATA24,SRSF4,SYDE1,UBC,USP24,US P35,USP40,USP27X,WBSCR27,ZNF480*
10	Cell Signaling, Nucleic Acid Metabolism, Small Molecule Biochemistry	18	13	*ADCY,ADGRA3,AMPK,ARHGAP26,Calmodulin,CD3,Collagen(s),Creb,DAB1,EPAS1,F ocal adhesion kinase,GNAL,GNPDA1,GNRH,Gpcr,GPR22,Histone h3,IL12 (family), Mapk,MC2R,MC5R,OPRD1,p85 (pik3r),PAIP2,PIK3CG,Pka,Pkc(s),Rac,Ras,Ras*

				homolog,RXFP2,SRC (family),TCR,tubulin (complex),Vegf
11	Developmental Disorder, Hereditary Disorder, Ophthalmic Disease	18	13	*ANO10,B3GALTL,C1orf168,CCDC138,CCDC71L,CNTLN,DUS4L,EARS2,FANCA,FDFT1,GK5,HDDC2,KDELC2,KMT2D,LDLRAD4,MARVELD1,NT5DC1,RAB42,RANGRF,REEP2,RELL1,RIMKLB,SLC35F5,SOHLH2,SYPL2,TFAP2D,TRIQK,TRMT2B,TRNAU1AP,TRPV2,TTC7A,UBC,UBFD1,UBL3,ZNF761*
12	Organismal Injury and Abnormalities, Digestive System Development and Function, Organ Morphology	14	11	*C5,C5-C6-C7-C8,C5-C6-C7-C8-C9,C8A,C8B,C8G,CCND1,CEP95,CLEC6A,CXCL11,DDIAS,DONSON,ENDOD1,FAM210A,Fcr,GLIS1,IDO2,IFNG,IL22RA2,KIF20A,LRRC4,LRRTM2,MTUS2,NLRP6,NXF1,PGLYRP2,PGLYRP3,PGLYRP4,PRELP,RAB20,SETD5,SLC16A9,SLC23A1,YIPF1,ZNF324*
13	RNA Post-Transcriptional Modification, Cellular Assembly and Organization, Cancer	14	11	*ANAPC1,ANAPC2,ANAPC5,ANAPC7,CALM1 (includes others),CDC16,CDC23,CPSF1,CPSF2,CPSF3,CYFIP2,DCLK1,DMAP1,H2AFB3 (includes others),H2AFZ,HNRNPR,INCENP,KDM3B,MATR3,NCKAP1,PAICS,PDCD7,PHACTR4,RAB6B,RFC2,RFC3,RFC4,RFC5,RYR1,SMARCAD1,SYMPK,USP20,VAT1L,WDR33,WWOX*
14	Organismal Development, Organismal Functions, Carbohydrate Metabolism	11	9	*26s Proteasome,AACS,ACSM5,ADIPOQ,Adipor,ANO9,Anti-inflammatory Cytokine,ATP5G2,C1QTNF5,C2orf61,Cyp2j9,DLG1,DUOX1,ETF1,FITM2,GPR39,GYS2,Il15r,Insulin,LBX1,LDLRAD1,LIG4,MAL,mir-375,NNAT,P38 MAPK,PARS2,POU5F1,PRKAA2,RNA polymerase II,RNMT,SCAF8,Tnf (family),TRPM2,ZNF462*
15	Cell-To-Cell Signaling and Interaction, Nervous System Development and Function, Cardiac Arrythmia	2	1	*MYBPC2,PCDH12*

Table S6. List of 45 lipid-related genes selected to study their expression in the present study. Genes are labelled with different color to indicate its overlapping between categories.

Selection of candidate genes	Article	Genes
Genes differentially expressed in *Longissimus dorsi* muscle of two phenotypically extreme groups of animals for intramuscular FA composition in the IBMAP cross	Puig-Oliveras *et al.*, 2014	*ACAA2, AQP7, ALB, ANGPT1, ATF3, MLXIPL, FOS, HIF1AN, PIK3R1, PLIN5, PPARG, SCD, SLC2A4, NFKB1, PPARGC1A*
Candidate functional and positional genes identified in a GWAS study for intramuscular FA composition in the IBMAP cross	Ramayo-Caldas *et al.*, 2012	*FABP5, PIK3R1, PLA2G12A, PPAP2A*
Genes related to lipid metabolism identified in gene co-association networks for FA composition in the IBMAP cross	Ramayo-Caldas *et al.*, 2014	*ACSM5, ANGPT1, FABP3, FABP5, MGLL, NCOA2, PEX2, PPARG, SETD7*
Genes related to lipid metabolism identified in gene co-association networks for fatness and growth related traits in the IBMAP cross	Puig-Oliveras *et al.*, 2014	*ALB, CREG1, ELF1, FABP5, MGLL, PPARG*
Lipid metabolism regulators (Transcriptional factors, cofactors and nuclear receptors)	Zhang *et al.*, 2012	*ETS1, LPIN1, NR1H3, NCOA1, NCOA6, PPARA, PPARD, PRKAA1, RXRG, SP1, SREBF1*
Enzymes involved in lipid metabolism		*ACSS1, ACSS2, CPT1B, CROT, DGAT1, DGAT2, PDHX*
Gene containing causal mutation of the imprinted QTL for muscle growth and fat deposition in a Meishan × Large White intercross	Van Laere *et al.*, 2003	*IGF2*

Table S7. Primers used for the ACSM5 SNP (rs331702081) genotyping by Taqman OpenArrayTM genotyping plates.

SNP	Gene	Full name	Primer sequence		Genotyping dye reporter	
			Forward	Reverse	VIC	FAM
rs331702081	*ACSM5*	acyl-CoA synthetase medium-chain family member 5	5'-CTGGAAGCCAAGCTCATTGG-3'	5'-AGCCCTCAGCCAGCAG-3'	5'-AGGACATGTGGCTTCAG-3'	5'-AGGACATGTGACTTCAG-3

Table S8. Primers used for the analyses of gene expression of the 48 genes by Real-Time PCR.

Gene	Full name	Type	Primer sequence	
			Forward	Reverse
ACAA2	acetyl-CoA acyltransferase 2	Target	5'-TGGATTGGATCTCAAGCTAGAAGA-3'	5'-CCATCGGGATTTGGATATGCT-3'
ACSM5	acyl-CoA synthetase medium-chain family member 5	Target	5'-TGTAATCTGTGCCAATCCCAAA-3'	5'-CATCATCTACGATCTGCACCTCAT-3'
ACSS1	acyl-CoA synthetase short-chain family member 1	Target	5'-TCAAGGGAGAAGCTGCGTTT-3'	5'-CCGACCGGAGCTCTTTCAC-3'
ACSS2	acyl-CoA synthetase short-chain family member 2	Target	5'-TGAAGCTGAAGGTTATCTGGTGTTC-3'	5'-CGTTCATGGTTCCCGTAGA-3'
ACTB	actin, beta	Endogenous	5'-CAAGGACCTCTACGCCAACAC-3'	5'-TGGAGGCGCGATGATCTT-3'
ALB	albumin	Target	5'-TCGTCGAGATACATACAAGAGTGAAA-3'	5'-AGCACTAGGCCTTTGAAATATTGTTC-3'
ANGPT1	angiopoietin 1	Target	5'-ACCAGCCTCCTCTCTCAAACC-3'	5'-GTTTGATTTAGTACCTGGGTCTCAACA-3'
AQP7	aquaporin 7	Target	5'-TTCAGCAGACATCTAACAATCTCAAAG-3'	5'-GACCTGGTAGACCGCCTCTTC-3'
ATF3	activating transcription factor 3	Target	5'-TCAGTCACGAAAGCCGAGGTA-3'	5'-GGCGGCAATCTTATTTCTTTCC-3'
CPT1B	carnitine palmitoyltransferase 1B (muscle)	Target	5'-ACATATCTACCTGTCCGGGATCA-3'	5'-CCCTGAGGATGCCATTCTTG-3'
CREG1	cellular repressor of E1A-stimulated genes 1	Target	5'-TAGGCAACTTGCAGGATAATCCA-3'	5'-ATCCATATTTCCTGCAGAAACTAGTCT-3'
CROT	carnitine O-octanoyltransferase	Target	5'-GGGAAACGAAATTGGTTGGA-3'	5'-CGCAAAGTTGACATTCAGTTGTG-3'
DGAT1	diacylglycerol O-acyltransferase 1	Target	5'-CCTGAATTGGTGTGTGGTCATG-3'	5'-GATGCCGTACTTGATGAGGTTCTC-3'
DGAT2	diacylglycerol O-acyltransferase 2	Target	5'-GGAACACGCCCAAGAAAGGT-3'	5'-GGATGGGAAAGTAGTCTCGAAAGTAG-3'
ELF1	E74-like factor 1 (ets domain transcription factor)	Target	5'-GATGACATCACCCTGACAGTTGA-3'	5'-TCCGCAGCCTCGATGGT-3'
ETS1	v-ets avian erythroblastosis virus E26 oncogene homolog 1	Target	5'-CCTGGGAATCCCAAAAGATCC-3'	5'-ACTCATTGACAGCCCACATCAC-3'
FABP3	fatty acid binding protein 3, muscle and heart	Target	5'-CGGGCACCTGGAAGCTAGT-3'	5'-GGCAAAACCCACACCAATTG-3'
FABP5	fatty acid binding protein 5 (psoriasis-associated)	Target	5'-CCAATGGAGAATTGGTTCAACA-3'	5'-GTTCATGACGCATACCACCACTA-3'
FOS	FBJ murine osteosarcoma viral oncogene homolog	Target	5'-CCGTCAATGCGCAGGACT-3'	5'-TCTGGGCTGGTCGAGATAGC-3'
HIF1AN	hypoxia inducible factor 1, alpha subunit inhibitor	Target	5'-TTGGCATGGAAGGAAATGTG-3'	5'-GCCTTTTATCTGAGCAAAGAAGTTCT-3'
HPRT1	hypoxanthine phosphoribosyltransferase 1	Endogenous	5'-TCATTATGCCGAGGATTTGGA-3'	5'-CTCTTTCATCACATCTCGAGCAA-3'
IGF2	insulin-like growth factor 2	Target	5'-GACCGTGCTTCCGGACAA-3'	5'-CGTTGGGCGGACTGCTT-3'
LPIN1	lipin 1	Target	5'-CCGAGAGAAGGTGGTGGACAT-3'	5'-CTCTCCATTGTCTCCCAGTTTCA-3'
MGLL	monoglyceride lipase	Target	5'-GTGTTCGCCCACGACCAT-3'	5'-CCTGACGAACACCTGGAAGTC-3'
MLXIPL	MLX interacting protein-like	Target	5'-CCCAAGTGGAAGAATTTCAAAGG-3'	5'-CTTCCTCCGCTCCACATACTG-3'
NCOA1	nuclear receptor coactivator 1	Target	5'-AAGGAACAGATGGATCCTTGTAACA-3'	5'-TGGTCAAGGTCAGCTGTAAACTG-3'
NCOA2	nuclear receptor coactivator 2	Target	5'-CGTACCCACACAGGCACCTAT-3'	5'-CTGTCGAAGGTGCTGGTTCA-3'
NCOA6	nuclear receptor coactivator 6	Target	5'-TTACATCCAGGCCTAGGAGGAAT-3'	5'-TTGCATAAAGTTCGGATTGGC-3'

NFKB1	nuclear factor of kappa light polypeptide gene enhancer in B-cells 1	Target	5'-CCCACAGACGTTCATAGACAATTT-3'	5'-GAGGCTGGTTTTGTAATGTTGACA-3'
NR1H3	nuclear receptor subfamily 1, group H, member 3	Target	5'-CTGGGCATGATCGAGAAGCT-3'	5'-TGGGCCAAGGCGTGACT-3'
PDHX	pyruvate dehydrogenase complex, component X	Target	5'-AGCTGTGGTTACCTTAGATGCAAGT-3'	5'-TTTTAGATCCTTCGGCAACCACTA-3'
PEX2	peroxisomal biogenesis factor 2	Target	5'-CTCAGACTCCTAAGAAACCTTCAGAGA-3'	5'-ACTGATTCTGAGCACTCTGTTTGC-3'
PIK3R1	phosphoinositide-3-kinase, regulatory subunit 1 (alpha)	Target	5'-TGGGGAGATATCTCGAGGGAA-3'	5'-TTTTAGTAGATGCGTCTCGTACCAA-3'
PLA2G12A	phospholipase A2, group XIIA	Target	5'-CCCACTCTTTGGTGTTCATCTTAA-3'	5'-ATAGCACCTGTCGTGCTGGTT-3'
PLIN5	perilipin 5	Target	5'-CTCAAACGATCCATGAGCCA-3'	5'-TGCCAGTGCCGCGAG-3'
PPAP2A	phosphatidic acid phosphatase type 2A	Target	5'-GGCCACTCTTCATTCTCCATGTAC-3'	5'-AGGCCCACGTAAATGGATACAG-3'
PPARA	peroxisome proliferator-activated receptor alpha	Target	5'-GGCACTGAACATCGAATGTAGAATC-3'	5'-CCGAAAGAAGCCCTTGCAA-3'
PPARD	peroxisome proliferator-activated receptor delta	Target	5'-GCATGTCTCA CAACGCCATT-3'	5'-GCTGACTCCCCTCGTTTGC-3'
PPARG	peroxisome proliferator-activated receptor gamma	Target	5'-TTGTGAAGGATGCAAGGGTTT-3'	5'-ATCCGACAGTTAAGATCGCACCTA-3'
PPARGC1A	peroxisome proliferator-activated receptor gamma, coactivator 1 alpha	Target	5'-CTCTGGAACTGCAGGCCTAA-3'	5'-TGGAGAAGCCCTAAAAGGGTTAT-3'
PRKAA1	protein kinase, AMP-activated, alpha 1 catalytic subunit	Target	5'-GTAAAAATGGAAGGCTGGATGAA-3'	5'-TGTGACAATAATCCACACCAGAAAG-3'
RXRG	retinoid X receptor, gamma	Target	5'-GAGGATTCTGGAAGCTGAACTTG-3'	5'-TCATTCGTCGAATTCTCCATGT-3'
SCD	stearoyl-CoA desaturase (delta-9-desaturase)	Target	5'-GGTGATGTTCCAGAGGAGGTACTAC-3'	5'-CAGCAATACCAGGGCACGAT-3'
SETD7	SET domain containing (lysine methyltransferase) 7	Target	5'-TGCTGGATATACTACCCAGATGGA-3'	5'-TCTCCTGTCATCTCCCCATCTT-3'
SLC2A4	solute carrier family 2 (facilitated glucose transporter), member 4	Target	5'-TCTGTGGGTGGCATGTTCTC-3'	5'-GAACAGCATTGCCTTCTTCCTT-3'
SP1	Sp1 transcription factor	Target	5'-CCACCATGAGCGACCAAGA-3'	5'-GCCACCAACTCCTTTTTCAATC-3'
SREBF1	sterol regulatory element binding transcription factor 1	Target	5'-CACGGAGGCGAAGCTGAATA-3'	5'-GCTTCTGGTTGCTCTGCTGAA-3'
TBP	TATA box binding protein	Endogenous	5'-CAGAATGATCAAACCGAGAATTGT-3'	5'-CTGCTCTGACTTTAGCACCTGTTAA-3'
